# Advancing *In Situ* Analysis of Biomolecular Corona: Opportunities and Challenges in Utilizing Field-Flow Fractionation

**DOI:** 10.1021/acsbiomedchemau.4c00001

**Published:** 2024-03-21

**Authors:** Soheyl Tadjiki, Shahriar Sharifi, Afsaneh Lavasanifar, Morteza Mahmoudi

**Affiliations:** † Postnova Analytics Inc., Salt Lake City, Utah 84102, United States; ‡ Department of Radiology and Precision Health Program, Michigan State University, East Lansing, Michigan 48864, United States; § Faculty of Pharmacy and Pharmaceutical Sciences, University of Alberta, Edmonton, Alberta T6G 2H7, Canada; ∥ Department of Chemical and Material Engineering, University of Alberta, Edmonton, Alberta T6G 2 V4, Canada

**Keywords:** biomolecular corona, *in situ*, biological fluids, field-flow fractionation, nanoparticle, nanomedicine, robustness, soft corona, hard corona

## Abstract

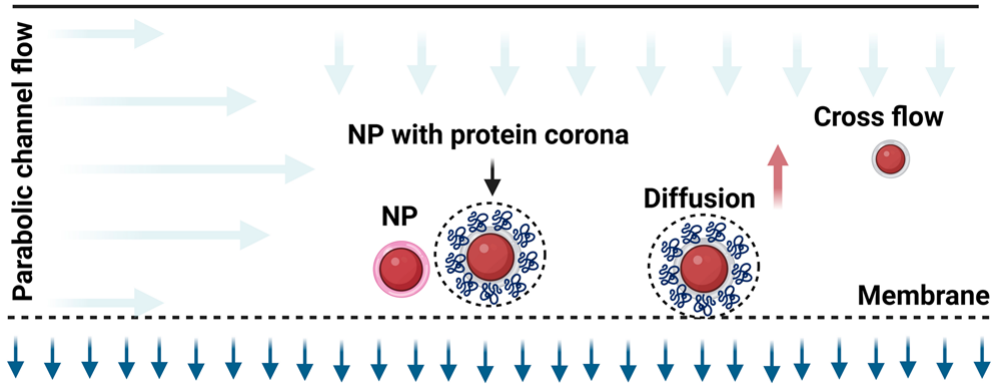

The biomolecular corona, a complex layer of biological molecules, envelops nanoparticles (NPs) upon exposure to biological fluids including blood. This dynamic interface is pivotal for the advancement of nanomedicine, particularly in areas of therapy and diagnostics. *In situ* analysis of the biomolecular corona is crucial, as it can substantially improve our ability to accurately predict the biological fate of nanomedicine and, therefore, enable development of more effective, safe, and precisely targeted nanomedicines. Despite its importance, the repertoire of techniques available for *in situ* analysis of the biomolecular corona is surprisingly limited. This tutorial review provides an overview of the available techniques for *in situ* analysis of biomolecular corona with a particular focus on exploring both the advantages and the limitations inherent in the use of field-flow fractionation (FFF) for *in situ* analysis of the biomolecular corona. It delves into how FFF can unravel the complexities of the corona, enhancing our understanding and guiding the design of next-generation nanomedicines for medical use.

## Introduction

The biomolecular corona effectively determines the identity of nanoparticles (NPs) in a biological environment.^1^ The formation of biomolecular corona can significantly alter the physical and chemical properties of the NP (e.g., size, charge, and hydrophobicity).^2^ These changes can affect the NP’s stability, aggregation, and circulation time in the body.^3^ Therefore, a thorough understanding of the biomolecular corona and its effects on the physicochemical properties and colloidal stability of NPs can guide their design to minimize adverse effects, making them safer for clinical applications.^4^

Traditionally, several methods such as liquid chromatography–tandem mass spectrometry and gel electrophoresis were used to be utilized to determine the corona composition.^5^ These methods commonly relay on an assumption that the NPs in each population behave similarly as far as protein adsorption to their surface is concerned and provide an averaged determination of corona composition for a given NP population.

While various techniques, such as centrifugation, have been employed to isolate hard corona-coated NPs,^6^ achieving a comprehensive *in situ* analysis of the biomolecular corona remains a significant challenge in the field.^7,8^ The term “*in situ* biomolecular corona” refers to the corona formed on the surface of NPs in the presence of excess proteins and biomolecules. The analysis of the hard corona, which involves removing excess and loosely bound proteins, is crucial for understanding the biological identity of NPs. However, this approach faces two primary obstacles: (i) techniques used may inadvertently introduce protein contaminants into the biomolecular corona outcomes, potentially skewing the results;^9^ and (ii) more importantly, these methods often overlook the role of the soft corona. The soft corona, comprising dynamically bound proteins, plays a pivotal role in modulating NP-cell interactions.^8^ Neglecting soft corona in studies is a notable oversight, as it can significantly impact how NPs are recognized and processed in the biological systems.^7,8^

In contrast, *in situ* analysis of the biomolecular corona provides a more realistic representation of the *in vivo* environment. This approach enhances our ability to predict the behavior of NPs within the human body more accurately.^7,8^ By gaining a deeper and more precise understanding of the *in situ* biomolecular corona composition of NPs, the nanomedicine community can better ascertain how biosystems, including various cells and tissues, recognize and interact with these particles. This knowledge is critical in understanding of influential factors in NP behavior in the biological systems including NPs’ cell uptake, tissue distribution, and subsequent cellular responses, thereby advancing the field of nanomedicine.

**Figure 1:**
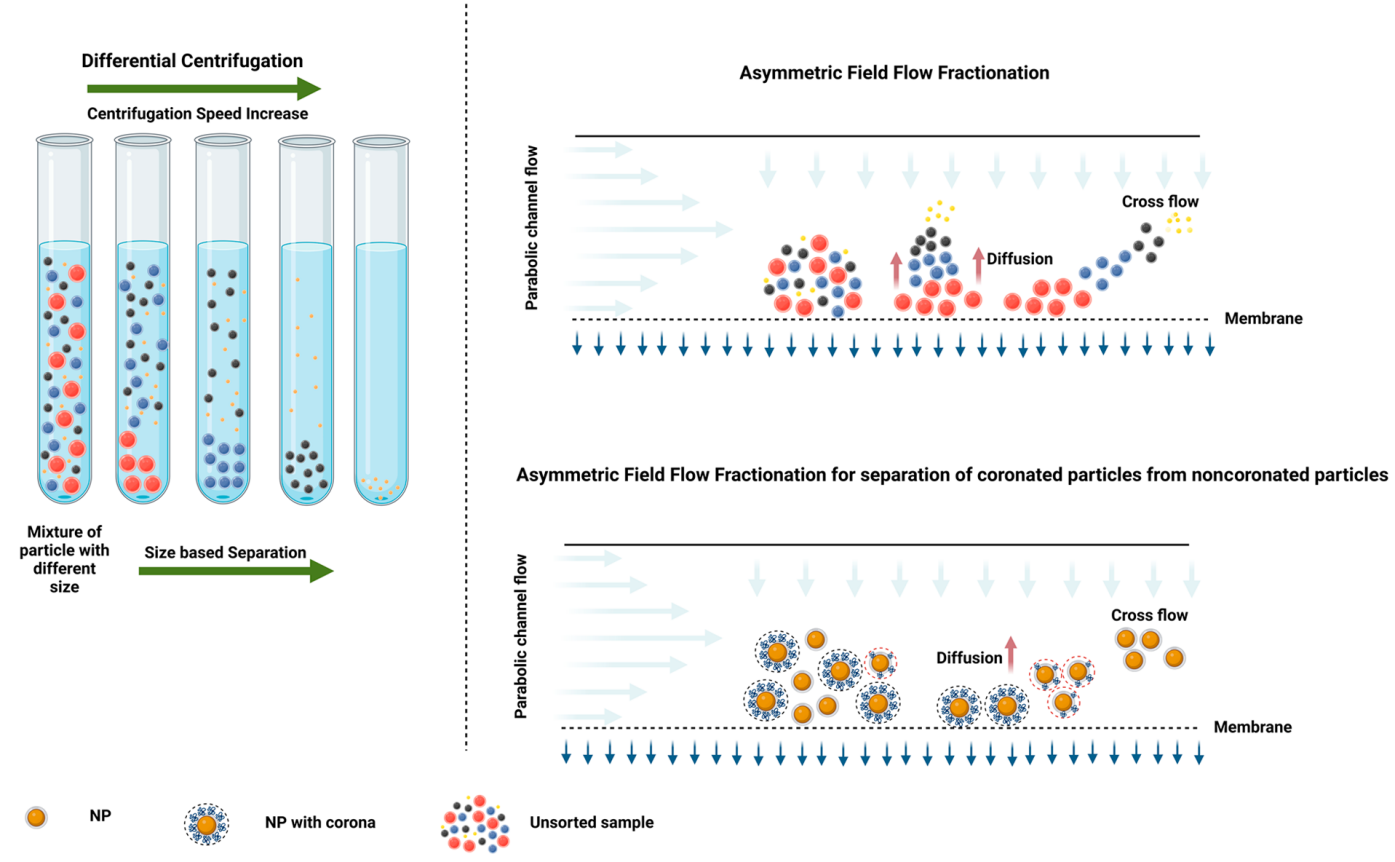
Scheme presents the primary methodologies employed for extracting coronated NPs from biological fluids. On the left, the differential centrifugation technique is shown, which relies on successive rounds of centrifugation and washing to separate NPs from biological matrices. The right side of the figure illustrates a sophisticated approach: asymmetrical flow field-flow fractionation (AF4). This graphic also showcases the application of these techniques in isolating coronated NPs for investigating both hard and soft protein coronas. Image created using Biorender, 2023.

A few techniques have been developed/adapted and utilized for *in situ* analysis of the biomolecular corona on NPs. Among them, differential centrifugal sedimentation (DCS) stands out as one of the earliest and most effective methods. Differential centrifugal sedimentation is a method originally used for the separation of cell organelles based on their sedimentation velocity under sequentially increasing centrifugation speeds (Figure 1). Equilibrium density-gradient centrifugation is a more fine-grained version of the test, where a density dependent separation profile is made using solutions of different gradient densities.^10^ DCS can be utilized to correlate the sedimentation kinetics of NPs with biomolecule adsorption on their surface. DCS is particularly renowned for its ability to provide high-resolution quantitative data regarding the thickness and uniformity of the biomolecular corona. This level of detail is essential for a comprehensive understanding of NP behavior in biological systems.^11,12^ Moreover, DCS offers the advantage of rapid sample processing, which significantly enhances efficiency.^13^

Despite its effectiveness, DCS does present several challenges that need to be carefully considered: (i) Sample integrity concerns: the application of high centrifugal forces in DCS could potentially alter NP-protein interactions; this may result in the loss or rearrangement of the corona, thereby affecting the integrity of the sample. (ii) Sensitivity to NP density: DCS’s accuracy is dependent on the density of the NPs being analyzed. Misinterpretation of results can occur if there is a failure to properly account for variations in the NP density. In addition, the *effective* density of particles at nanoscale regime is dramatically different from their actual density^14^ which creates further complications in accurate and robust analysis of corona coated NPs. (iii) Calibration requirements: the technique typically necessitates calibration using standard particles of known size and density. While this ensures precision, it can also pose limitations, particularly in scenarios where standard particles are not readily available or do not accurately represent the sample being studied. These challenges highlight the need for careful implementation and interpretation of results when using DCS in the analysis of the biomolecular corona on NPs. Despite these hurdles, DCS remains a valuable tool in the field, offering insights that are critical for advancing our understanding of NP interactions within biological environments.

Cryogenic transmission electron microscopy (Cryo-TEM), a variant of transmission electron microscopy (TEM), allows for the observation of samples under cryogenic conditions, typically using liquid nitrogen temperatures (−196 °C or 77 K).^15−18^ This method is exceptionally valuable in structural biology and nanotechnology. A critical application of Cryo-TEM is in the direct visualization of biomolecular coronas on NPs, offering insights into their intricate structures.^9,19^ A key benefit of Cryo-TEM over conventional TEM lies in its capacity to observe samples in a hydrated state. This feature is crucial for biological specimens as it more accurately reflects biological structures in their natural environment. Additionally, Cryo-TEM eliminates the need for staining or fixing samples, processes that can modify their original states. Consequently, Cryo-TEM facilitates a detailed examination of the structural nuances of the corona.^9^ However, despite these significant advantages, Cryo-TEM requires careful sample preparation to prevent the formation of artifacts. Moreover, the method primarily provides qualitative data and lack of wide number of NPs evaluation (mainly due to the existence of limited numbers of images), which underscores the need for precision in its application and interpretation.^9^ One strategy to overcome this major issue would be the development of automated imaging and image analysis to satisfy the required meaningful statistical analysis of a large number of NPs.

For specific NP types, such as metallic ones, surface plasmon resonance (SPR) is an effective method for real-time, label-free analysis of biomolecular corona formation.^20,21^ In a typical SPR analysis, NPs are incubated with the biological fluid of interest (e.g., plasma), isolated by centrifugation, and then resuspended in buffer. It is important to note that in the *in situ* analysis of biomolecular corona using SPR, the step involving the isolation of NPs through centrifugation should be omitted. This suspension is then flowed, in the SPR channels, onto surfaces immobilizing suitable ligands, such as antibodies against potential adsorbed protein such as human serum albumin (HAS) or Apo (HSA-Ab and ApoE-Ab, respectively). SPR has become an invaluable tool in improving the analysis of biomolecular coronas, particularly in the study of NP interactions with biological molecules, as (i) it allows for the real-time observation of biomolecular interactions, (ii) unlike many other techniques, SPR does not require labeling of the proteins or NPs, (iii) it has high sensitivity to changes in mass at the surface of a sensor chip, (iv) it not only detects the presence of proteins binding to NPs but also provides quantitative data on the kinetics and affinity of these interactions, (v) it characterizes how different environmental conditions (e.g., pH, temperature, or ionic strength) affect the formation of the biomolecular coronas, (vi) it can be used with a range of biological fluids, such as blood, plasma, serum, or even cell culture media, and (vii) it can analyze multiple samples simultaneously, making it an efficient tool for high-throughput screening.^20,22−24^ One of the major issues of the SPR approach for biomolecular corona analysis is the complexity of its setup, which is highly sensitive to experimental conditions and may lead to potential misinterpretations of results. Furthermore, SPR is a time-intensive technique requiring a high level of expertise for accurate data interpretation.

In the realm of *in situ* biomolecular corona analysis, field-flow fractionation (FFF) has emerged as a particularly promising technique.^25^ FFF uniquely addresses many of the limitations inherent in other methods. While it has its own set of challenges, FFF’s adoption in the analysis of the biomolecular corona is replete with potential. It offers a pathway toward more precise and comprehensive characterizations of NPs within biological systems. This level of detail is vital for the advancement and clinical application of nanomedicine, enhancing the likelihood of successful translation from laboratory research to clinical use. Furthermore, FFF addresses the limitations associated with protein contaminations^4,9,26^ that can challenge traditional methods of analyzing the biomolecular corona, such as liquid chromatography–tandem mass spectrometry (LC-MS/MS) and gel electrophoresis. FFF provides precise information on the biomolecular corona’s composition by enabling the collection of corona-coated NPs without contamination. It is crucial to understand that the issues of protein contamination in LC-MS/MS and gel electrophoresis primarily arise from the nonrobust collection methods of corona-coated NPs, rather than the analytical techniques themselves.^26−28^ Specifically, FFF facilitates the isolation of clean, contamination-free corona-coated NPs, allowing for the accurate analysis of the biomolecular corona by using LC-MS/MS and gel electrophoresis.

## Fundamentals of Field-Flow Fractionation

FFF is a distinct member of the liquid chromatography techniques, a groundbreaking invention by Giddings in 1966.^29^ Unlike traditional chromatography methods, FFF stands out for its unique separation mechanism that occurs in a column devoid of a stationary phase or, more precisely, in an open channel. In conventional size-exclusion-based chromatography, the column is filled with a porous material. Smaller particles diffuse into this material and traverse a longer path, resulting in a slower elution. Conversely, larger particles are excluded from these pores and elute more quickly, setting a separation order from large to small particles.

FFF, however, operates on a different principle. It employs a long, slender, open channel, typically ranging from 10 to 30 cm in length but only a few hundred microns thick. The flow within this channel is laminar, meaning the flow speed is zero at the walls and reaches its maximum at the channel center. Instead of a stationary phase, FFF utilizes a physical field perpendicular to the flow direction and spanning the smallest channel dimension (its thickness). The physical field used in FFF can vary depending on the specific type of FFF technique being employed; for example, asymmetric flow-, sedimentation-, thermal-, electric-, and magnetic-FFF use a flow-, centrifugal-, thermal-, electric-, and magnetic-field.

Upon introducing a colloidal sample into the channel, the field exerts a force on the particles, driving them toward the channel bottom. This movement is counteracted by diffusion of the particles. An equilibrium is established when the rates of field-induced migration and diffusion balance each other. At this equilibrium, particles of different sizes form “clouds” with varying thicknesses–smaller particles form a broader, less compressed cloud, moving faster, whereas larger particles create a denser, thinner cloud, moving slower through the channel.

Thus, the elution order in FFF is the reverse of size exclusion chromatography (SEC): smaller particles are eluted first, followed by larger ones. The absence of a stationary phase in FFF not only makes it a low-pressure and low-shear technique, earning it the signature of “gentle separation,” but also ensures the preservation and accurate characterization of delicate samples like aggregates or agglomerates. This gentle yet effective separation process is pivotal for analyzing complex samples without altering their native states, an essential feature for many applications in the fields of biochemistry and nanomedicine.

## Field-Flow Fractionation Subtechniques

Various subtechniques of FFF are distinguished by the type of external field applied during the separation process. Each subtechnique utilizes a specific field to achieve particle separation, and these are tailored to measure different particle parameters. A comprehensive summary of these FFF subtechniques, detailing the specific fields employed and the corresponding particle parameters that can be measured, is provided in Table 1 for comparison.

**Table 1:** Different FFF Subtechniques, Applied Fields Employed, and Measured Parameters in the Brownian Mode

FFF subtechniques	applied field	measured sample property
centrifugal	centrifugal	buoyant mass
asymmetrical flow	cross flow	diffusion coefficient
thermal	thermal gradient	molar mass; surface composition
electrical	electric	charge; electrophoretic mobility
magnetic	magnetic	magnetophoretic mobility; size
acoustic	acoustic	compressibility; size; density
dielectrophoretic	dielectrophoresis	dielectrophoretic mobility

Among the various FFF subtechniques, asymmetrical flow FFF (AF4) stands out as the most universally adopted and widely used.^30^ Given its prominence and widespread application, here we will specifically focus on detailing the AF4 subtechnique to provide a comprehensive understanding of its operation and applications.

## Asymmetrical Flow Field-Flow Fractionation

In AF4, the separation process is driven by a cross-flow field applied perpendicularly to the channel flow, which carries the sample species. The channel itself is uniquely designed, sandwiched between a nonpermeable wall on one side and a semipermeable wall on the other. This semipermeable wall permits some carrier fluid to traverse the channel, thereby establishing the necessary crossflow. To prevent sample loss, an ultrafiltration membrane is placed over the accumulation wall.

The cross section of the AF4 channel is typically trapezoidal, with a solid Lucite or glass plate replacing the upper semipermeable wall. When the sample enters the channel, it encounters two distinct flow paths: (i) the channel flow, which proceeds along the length of the channel and eventually carries the separated sample species to the detector; and (ii) the field flow, which exits through the lower membrane and the semipermeable wall.

In the AF4 technique, the process of sample focusing plays a pivotal role in delivering sample species into the separation channel. Throughout this phase, the sample species interact with the exerted field, forming equilibrium zones or clouds that exhibit varying average thicknesses. Such differentiation enables these zones to be transported at distinct velocities during the elution phase that follows. This mechanism is fundamental to the separation efficiency of AF4, allowing for the precise analysis of complex mixtures based on size and shape.

During sample injection, a unique focusing zone is created between the inlet and outlet channel ports. This is achieved by introducing two unbalanced and counter-flowing streams, which are critical in establishing the sample’s position within the channel. The flow rate ratio of these counter-flowing streams determines the location of the focusing zone. Specifically, the flow carrying the sample plug is slower than its counterpart, positioning the focusing zone nearer the sample injection port. To ensure optimal separation, the sample plug requires a finite period (usually a few minutes) to reach the focusing zone. This transit time is crucial to focus the sample into a thin line against the accumulation wall and to achieve equilibrium, thereby reducing band broadening (plate height) and enabling the injection of large sample volumes.

## Asymmetrical Flow Field-Flow Fractionation: Integration with Multidetection Systems

The integration of AF4 with a multidetection system, which includes both static and dynamic light scattering, provides comprehensive insights into particle size and molecular weight. This combination offers a unique advantage in gathering detailed information about the particle shape. Dynamic light scattering, in particular, yields critical parameters such as the hydrodynamic radius (Rh), while static light scattering provides parameters such as the radius of gyration (Rg). These metrics are instrumental in characterizing the particle morphology.

The Rg is especially significant as it reflects the mass distribution within a particle, shedding light on its internal structure and degree of flexibility. For example, when analyzing polymeric NPs used in drug delivery, comparing Rg values can help researchers determine whether the macromolecules possess a compact, globular conformation or an extended, more flexible structure. Such insights are crucial to understanding the interaction of these NPs with biological systems and their consequent effectiveness.

Furthermore, the Rh obtained from dynamic light scattering offers valuable insights into the size of particles in a solution, taking into account factors such as hydration and shape. The comparative analysis of Rg and Rh values is crucial for understanding the shape characteristics of particles. In the realms of polymer science and biotechnology, this analysis enables researchers to differentiate between various particle shapes, such as spherical, rodlike, or irregular structures.

It is noteworthy that always caution must be taken when using the Rg/Rh ratio for determining particle shape, especially in the context of analyzing a diverse mixture of biological NPs using AF4. The Rg/Rh ratio can indeed provide insights into the shape and conformation of particles in solution; however, its application becomes complicated by a broad mixture of biological entities.

Biological NPs often exhibit a wide range of physical and chemical properties due to their diverse origins and compositions. When such a heterogeneous mixture is subjected to AF4, particles of similar sizes but different biological characteristics can coelute, leading to overlapping peaks. This phenomenon underscores the inherent challenge of using AF4 for shape analysis without considering the potential for coelution of biologically distinct species with similar hydrodynamic properties.

Understanding the shape of particles is critical for customizing the design of materials and nanocarriers across a range of applications. Whether it is enhancing the efficacy of drug delivery systems or optimizing the functionality of biomaterials in biomedical and industrial settings, shape-related data plays a pivotal role. The integrated examination of size, molecular weight, and shape through AF4 coupled with a multidetection system offers a holistic approach. This comprehensive methodology significantly enhances our understanding of the complexities inherent in particulate systems.

The integration of AF4 with dynamic and static light scattering, as well as concentration detectors such as refractive index (RI) and ultraviolet (UV) detectors, enriches the analysis of lipid NPs. While static light scattering provides the absolute molecular weight of particles, crucial for comprehending the composition and stability of lipid NPs, the concentration detectors bring an added dimension of precision. Specifically, RI and UV detectors play a vital role in quantitatively assessing the concentrations of various components within the lipid NPs. This capability is integral when AF4, as it allows for accurate measurement of the loading mass of therapeutic agents within lipid NPs, a key factor in drug delivery applications. The RI detector is sensitive to changes in the RI and is particularly adept at detecting variations in the lipid composition. On the other hand, the UV detector is tuned to specific wavelengths corresponding to the therapeutic payload, thus enabling the targeted monitoring of drug load.

Given the renowned sensitivity and specificity of fluorescence detectors in tracking nanocarriers and biomolecular movements, incorporating such a detector into the multidetection segment of the system is suggested. This addition would enhance the capability of monitoring fluorescent nanocarriers, enabling precise differentiation from biomolecules.

The integration of AF4 with a multidetection system is also of significant importance in analyzing biomolecular corona formation for several key reasons:

AF4 is a powerful separation technique that can fractionate particles based on their size and shape under a gentle flow condition. This is especially useful for studying biomolecular coronas. The ability of AF4 to separate these complex mixtures without altering their native state is crucial for accurate analysis.

By interfacing AF4 with multidetection systems, such as light scattering detectors (dynamic and static), and mass spectrometry, researchers can obtain a comprehensive set of data about the separated components. This includes information about their size, shape, molecular weight, composition, and chemical properties. Such detailed characterization is vital for understanding how the biomolecular corona influences the behavior and fate of NPs in biological systems.

The combination of AF4 with advanced detection methods enhances the sensitivity and specificity of the analysis. It enables the detection of even small changes in the corona composition, which could significantly affect the NPs’ interactions with cells and biological molecules. This is essential for investigating the mechanisms of NPs biointeractions and their potential effects, including toxicity, cellular uptake, and immune response.

The data obtained from AF4 coupled with multidetection systems can guide the design of NPs to improve targeting specificity, reduce off-target effects, and enhance therapeutic efficacy.

By combining these diverse detectors with AF4, researchers can also gain a comprehensive understanding of a wide range of NPs including clinically relevant lipid NPs formulations. This holistic approach not only assists in optimizing drug encapsulation efficiency but also contributes significantly to the development of more effective and precisely targeted drug delivery systems. Such advancements are particularly relevant in the field of personalized medicine, where tailored therapeutic solutions are essential.

## Asymmetrical Flow Field-Flow Fractionation: Determining *In Situ* Thickness of Biomolecular Corona

For the *in situ* measurement of the biomolecular corona thickness, NPs are introduced into a channel with asymmetric flow both with and without biological fluids in separate runs. The channel has a laminar flow profile, with the flow velocity being higher at the center and decreasing toward the walls. When NPs are introduced without biological fluids, AF4 characterizes the size of the bare NPs. Conversely, when NPs are introduced in the presence of biological fluids, AF4 effectively separates the corona-coated particles from excess biomolecules (Figure 1). This separation is facilitated by the increased size of the NPs resulting from the adsorption of biomolecules, forming the corona.

Remarkably, AF4 demonstrates the ability to distinguish species with as minimal as a 30% difference in their diffusion coefficients, showcasing its high resolution for analyzing complex mixtures characterized by multimodal distributions.^31^ This capability facilitates the detection of nuanced variations in the size and shape of particles or molecules. Nonetheless, it is critical to acknowledge that this impressive resolution might not always lead to the separation of different species into distinct, well-defined peaks. This constraint implies that although AF4 is highly suitable for specific applications, it may not serve as a universal solution for all analytical tasks that demand the differentiation of closely related species.

The crossflow in AF4 causes NPs and free biomolecules to separate based on their hydrodynamic radius. Corona coated NPs with a larger hydrodynamic radius will be closer to the accumulation wall, while smaller biomolecules (e.g., proteins) will be further away. This separation allows for the fractionation of corona coated NPs from excess biomolecules. If some NPs undergo colloidal instability and form aggregates during the formation of the biomolecular corona, then the FFF system is capable of detecting these aggregates. This detection is possible due to FFF’s sensitivity to changes in particle size and distribution, allowing it to distinguish between individual NPs and their larger aggregated counterparts. This comparative analysis allows for an indirect but accurate measurement of the corona thickness by assessing the size difference of the colloidal stable NPs before and after exposure to the biological fluids.

In addition, FFF can determine the shape of the formed biomolecular corona at the surface of NPs through the use of multiangle light scattering (MALS). This determination is based on calculating the ratio of Rg (obtained from MALS) to Rh (derived from dynamic light scattering) for each segmented sample. This ratio is an indicator of particle shape; it increases with the nonsphericity of the particles, starting from 0.775 for homogeneous spheres.^32,33^ For particles with an elliptical shape, their rotational aspect ratios can be deduced.

## Asymmetrical Flow Field-Flow Fractionation: Enabling the *In Situ* Isolation of Pure Biomolecular Corona

The AF4 process adeptly “isolates” NPs with a distinct thickness of biomolecular corona, distinguishing them from the remainder of the sample. This precise and selective fractionation is key to accurately representing the true composition of proteins associated with the corona. Without the use of AF4, traditional analytical techniques, such as liquid chromatography–tandem mass spectrometry and gel electrophoresis, might fail to accurately depict this composition. They tend to provide averaged information from billions of NPs, each potentially featuring varying compositions of the biomolecular corona.

In contrast, the utilization of AF4 allows for the isolation of specific corona-coated NP fractions. This isolation enhances our understanding of the functional roles of the biomolecular corona with respect to the NP population. Such detailed insight is crucial, as the nature of the corona substantially influences the outcomes of *in vitro* and *in vivo* experiments, and by extension, the results of numerous clinical trials. Therefore, AF4 stands out as an invaluable tool in nanomedicine research, offering a more nuanced and accurate analysis of the biomolecular corona and its impact on NP behavior in biological systems.

While the combination of AF4-MALS is a powerful technique for determining the size, shape, and molecular weight distribution of NPs in a sample, it is important to recognize that this method is not species-specific. AF4 separates particles based on their size and hydrodynamic properties, and MALS provides detailed information on their physical dimensions by measuring light scattering at multiple angles. However, this setup does not inherently distinguish between particles based on their chemical composition or biological identity. This limitation underscores the necessity of integrating other analytical methods that can offer species-specific information, such as spectroscopic techniques or mass spectrometry, to complement the data obtained from AF4-MALS.

Traditionally, centrifugation techniques have been used for the separation of coronated NPs from biological fluids. However, the specificity and accuracy of this method highly depend on several processing factors and more importantly on the composition of the NP corona, especially soft corona and hard corona. One critical factor in the separation of NPs from biological fluids is preservation of the corona composition without disrupting the corona composition by the experimental process. Centrifugation based process, however, utilizes several washing steps that can either remove some of the soft corona or result in overestimation of highly abundant proteins as the washing process may not be effective in removing all the adsorbed proteins.^34^

**Figure 2:**
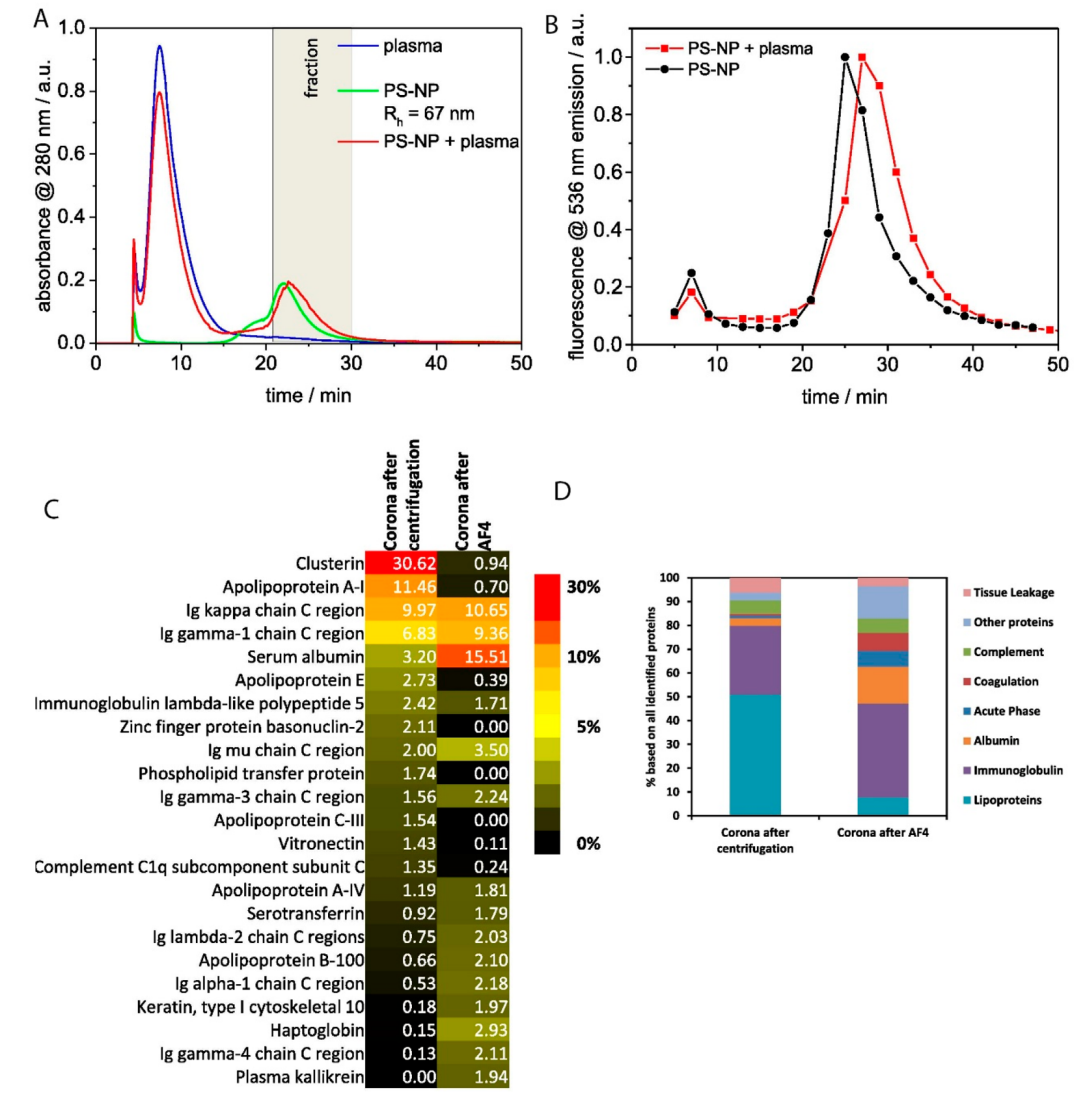
(A) This panel illustrates the asymmetrical flow field-flow fractionation (AF4) elution profiles for polystyrene NPs (PS-NPs) shown in green, human plasma in blue, and the red profile representing their combined incubated mixture. The gray box indicates the fraction collected after injecting the mixture, with the separation process carried out at 37 °C. (B) The accompanying elution graph displays the offline fluorescence signal from the runs detailed in panel (A). (C,D) These panels detail the protein composition of various protein coronas, as analyzed using liquid chromatography–mass spectrometry (LC-MS). The proteins are categorized based on their functions, and a heat map is provided to compare the abundance of individual proteins in protein coronas obtained through centrifugation versus the AF4 separation method. Reproduced or adapted with permission from ref (36). Copyright 2018 Elsevier.

Several studies have used FFF to investigate the protein corona formation on NPs. In one study by Ashby et al., human serum depleted albumin and IgG were incubated with SPIONs and the formation of protein corona was evaluated using AF4 and ultracentrifugation.^35^ The authors showed that both techniques can effectively separate the NP with hard corona from biological fluids. However, AF4 removed the weakly bounded proteins from NPs; therefore, NPs with soft corona could not be investigated using this technique. On other hand, another study by Weber et al. showed that formation of protein corona and especially soft corona on PEGylated polystyrene NPs incubated in human blood plasma can be evaluated using AF4 technique.^36^ Their method allowed separation of PS NPs from protein corona coated PS NP (Figure 2A,B), defining HSA as the major corona protein after AF4 separation, indicating that soft protein corona can be preserved during the separation in the AF4 technique (Figure 2 C,D). Authors also showed that both centrifugation technique and AF4 can preserve the hard corona. In another study, Alberg et al. used AF4 technique to analyze the protein corona formation and its composition on several core cross-linked NPs consisting of poly(*N*-2-hydroxypropylmethacrylamide) (pHPMA), polysarcosine (pSar), or poly(ethylene glycol) (PEG) as the shell forming block.^37^ Authors followed the following steps in their analysis. Initially, NPs were exposed to complete human blood plasma for 1 h at 37 °C followed by separation of NPs from unattached proteins with AF4. Particles were analyzed by multiangle light scattering and SDS PAGE and label-free quantitative proteomic analysis to evaluate the protein corona formation on the surface of NPs. Authors reported that particles separated using AF4 had 126 proteins for p(Sar)-NPs, 146 for p(HPMA)-NPs, and 128 for PEG-NPs with human serum albumin (HSA) as the predominant protein in the AF4 fractions across all particles. Interestingly, results also showed that the protein composition of NPS was not significantly enriched compared to plasma without particles in AF4. These results also indicated that the AF4 has the potential to separate the NPs with a soft corona. However, the results are strongly dependent on the composition of NPs, as some PEGylated NPs may have limited interaction with biological fluids or have a high dissociation adsorption rate with proteins.

## Future Directions and Conclusion

The use of field-flow fractionation (FFF) for studying the biomolecular corona presents a significant opportunity to advance our understanding of NP-biological interactions. Especially asymmetrical flow field-flow fractionation (AF4) has been shown to have good capability to fractionate and analyze NPs based on the thickness and composition of their biomolecular corona. By enabling the isolation of specific corona-coated NP fractions, AF4 deepens our understanding of the corona’s functional roles, influencing the outcomes of both experimental research and clinical trials. The use of FFF provides the following opportunities for *in situ* analysis of biomolecular corona: (i) FFF is a high-resolution technique that effectively separates NPs from unbound biomolecules, achieving fine separation with notable clarity; (ii) versatility: the technique is versatile in handling different types of NPs and biomolecular corona compositions, making it suitable for a broad range of applications; (iii) minimal sample perturbation: one of the biggest advantages of FFF is its gentle separation process, which minimizes the perturbation of the corona-NP interaction, thus maintaining the integrity of the sample; however there is still debate whether the FFF can preserve the formed soft corona on the surface of NPs; (iv) analytical complementarity: FFF can be coupled with other analytical techniques such as mass spectrometry, providing comprehensive information about the molecular makeup of the corona. Future developments could focus on enhancing the sensitivity and resolution of FFF, simplifying sample preparation methods and integrating computational tools for data analysis.

It is also worth noting that separation by FFF is highly dependent on the nature of the NPs and biological molecules. For example interaction of NPs with the FFF membrane can potentially complicate the separation process.^38^ In general, FFF may not be very useful in separation of weakly associated complexes such as soft protein coronas, as they may be disrupted during the process. The technique itself is complex and requires advanced knowledge of the existing and nature of interactions between NPs and biological molecules.

Hence, collaborations between interdisciplinary teams could drive the innovation of new FFF methodologies tailored to specific types of NPs and biological environments. In summary, the dynamic field of nanomedicine continues to evolve, and the *in situ* analysis of the biomolecular corona is at the forefront of this evolution. The ongoing refinement and integration of techniques such as AF4 will undoubtedly pave the way for groundbreaking advancements in nanomedicine, ultimately leading to improved therapeutic outcomes and realization of the full potential of nanomedicine technologies.
